# The Dynamic Role of FOXP3^+^ Tregs and Their Potential Therapeutic Applications During SARS-CoV-2 Infection

**DOI:** 10.3389/fimmu.2022.916411

**Published:** 2022-07-08

**Authors:** Zhan Xu, Xue Jiang, Xueyu Dai, Bin Li

**Affiliations:** ^1^ Center for Immune-Related Diseases at Shanghai Institute of Immunology, Department of Respiratory and Critical Care Medicine of Ruijin Hospital, Department of Immunology and Microbiology, Shanghai Jiao Tong University School of Medicine, Shanghai, China; ^2^ Department of Thoracic Surgery, Clinical Translational Research Center, Shanghai Pulmonary Hospital, Department of Integrated TCM and Western Medicine, Shanghai Skin Disease Hospital, Tongji University School of Medicine, Shanghai, China; ^3^ Institute of Arthritis Research, Guanghua Integrative Medicine Hospital, Shanghai University of Traditional Chinese Medicine, Shanghai, China; ^4^ Shenzhen Key Laboratory of Immunity and Inflammatory Diseases, Shenzhen, China

**Keywords:** Treg, FOXP3, COVID-19, SARS-CoV-2, Treg-based therapy

## Abstract

Coronavirus disease 2019 (COVID-19) has been raging all around the world since the beginning of 2020, and leads to acute respiratory distress syndrome (ARDS) with strong cytokine storm which contributes to widespread tissue damage and even death in severe patients. Over-activated immune response becomes one of the characteristics of severe COVID-19 patients. Regulatory T cells (Treg) play an essential role in maintaining the immune homeostasis, which restrain excessive inflammation response. So FOXP3^+^ Tregs might participate in the suppression of inflammation caused by severe acute respiratory syndrome coronavirus 2 (SARS-CoV-2) infection. Besides suppressive function, tissue resident Tregs are also responsible for tissue repair. In this review, we mainly summarize the latest research focusing on the change of FOXP3^+^ Tregs in the COVID-19 patients, discuss the relationship between disease severity and number change of Tregs and speculate the potential role of FOXP3^+^ Tregs during SARS-CoV-2 infection. Furthermore, we introduce some potential Treg-based therapies to improve patients’ outcomes, which include small molecular drugs, antibody drugs, CAR-Treg and cytokine treatment. We hope to reduce tissue damage of severe COVID-19 patients and offer better prognosis through Treg-based therapy.

## Introduction

Tregs are a CD4^+^ T cell subset which express high level of CD25 (high-affinity interleukin 2 receptor alpha) and forkhead box P3 (FOXP3) (1, 2). In the 1970s, Gershon and Kondo found that T cells owned suppressive function besides defense function through thymus-removed experiment on mice (3). Until the early of 2000s, researchers discovered IL-2 and its high affinity receptor CD25 were essential for the generation and maintenance of Treg subsets (4). In 2001, *foxp3* was firstly identified as an essential transcriptional factor gene for keeping immune homeostasis in the Scurfy mouse (5). In 2003, Sakaguchi and his team first brought up and proved that FOXP3 was essential to the development and function of Tregs (1). The mutation of FOXP3 will lead to immune dysregulation, polyendocrinopathy, enteropathy, X-linked syndrome (IPEX), along with severe autoimmune diseases like enteropathy, type 1 diabetes (T1D), dermatitis and other autoimmune diseases (6). Tregs are mainly categorized into two groups, one is natural Tregs (nTreg) which develop in thymus (7), the other is named as induced Tregs (iTreg) which are converted from naïve CD4^+^ T cells and could be generated both *in vivo* and *in vitro* (8). FOXP3^+^ Tregs are responsible for keeping immune tolerance, which can prevent allergic and other kinds of autoimmune diseases (9) as well as inhibit the anti-tumor or anti-pathogen immune responses (10).

Since the outbreak in 2020, the COVID-19 has raged all over the world. SARS-CoV-2 is mainly transmitted through respiratory droplets or aerosols (11). By 27th May 2022, SARS-CoV-2 had infected more than 527 million people and resulted in a death of 6.28 million people at least. The SARS-CoV-2 pandemic has led to a serious global public health crisis. Patients with COVID-19 exhibited the following symptoms including fever, dry cough, difficulty breathing, diarrhea, headache, myalgia and joint soreness (12, 13). The chest CT image of COVID-19 patients showed ground-glass opacity, and compared with healthy adults, the plasma concentrations of IL-1β, IL-7, IL-8, IL-9, IL-10, IFN-γ, TNF-α, and VEGF of COVID-19 patients were all upregulated (14). The damage of the SARS-CoV-2 to the human body is not only related to the viral infection, but also to the degree of the host immune response to the virus. The immune system released plenty of pro-inflammatory cytokines in response to the SARS-CoV-2 invasion, the uncontrolled inflammation would cause tissue damage in lung, heart, liver and kidney, which possibly leads to respiratory failure or multiple organ failure (15). Given the immune suppressive function and tissue repair ability of Tregs, Tregs are likely to provide protective functions and prevent cytokine storm. However, Tregs also possibly suppress innate and adaptive anti-viral immune responses. Consequently, Tregs may play dual roles during the process of SARS-CoV-2 infection.

## SARS-CoV-2 and Immune Response in COVID-19

SARS-CoV-2 has the characteristics of strong infectivity and high mutation rate. Since the outbreak, the virus has undergone several mutant strains, such as Delta (16), Omicron (17) and other variants. It has been reported that SARS-CoV-2 can severely impair the immune responses and lead to excessive inflammatory responses. SARS-CoV-2 belongs to the coronavirus family which is enveloped single-stranded, positive-sense RNA viruses, containing 28–34 kb genome (18). Genome of SARS-CoV-2 contains spike (S), envelope (E), membrane (M) and nucleocapsid (N) genes to encode the viral structural proteins and other open reading frames to encode non-structural proteins. During the invasion, S protein of SARS-CoV-2 binds to angiotensin-converting enzyme 2 (ACE2) of host cells (13). Other mild human coronaviruses like 229E, NL63, OC43 and HKU1 only infect the upper respiratory tract and cause mild symptoms (19). There also exists fatal human coronaviruses including severe acute respiratory syndrome coronavirus (SARS-CoV), Middle East respiratory syndrome coronavirus (MERS-CoV) and SARS-CoV-2 which can infect the lower respiratory tract and cause severe pneumonia (20). SARS-CoV-2 invasion to lung cells could lead to impaired respiratory function, creating a low blood oxygen and high lactate environment (21). Meanwhile, SARS-CoV-2 invasion would rapidly activate immune response as well.

The human immune system includes innate and adaptive immunity. Innate immunity acts as the first defender when virus invades host. When SARS-CoV-2 infects the host cells, the pattern recognition receptors, such as toll-like receptors (TLRs) and intracellular RNA sensors RIG-I, will recognize the invasion of foreign pathogens and induce downstream type I interferon (IFN) response program which is regarded as the most efficient cytokine to clear virus (22). Plenty of evidences indicate there exists strong cytokine storm in the COVID-19 patients, TNF-α and interleukins like IL-1β, IL-6, IL-8 are mainly secreted by the epithelial cells, endothelial cells, tissue macrophages and mast cells, the circulating levels of these cytokines increase acutely in COVID-19 patients (23, 24). The uncontrolled inflammatory responses excessively activate immune cells like T-cells, macrophages and natural killer cells, causing vascular endothelial damage, alveolar epithelial damage, diffuse alveolar damage, disseminated intravascular coagulation (DIC), multiorgan failure and even death in severe patients (25).

During the virus attack, T cells play an essential roles in the process of antiviral antibody production and cell-mediate immune response. Lymphopenia with reduced numbers of CD4^+^ and CD8^+^ T cells is an obvious symptom in severe COVID-19 patients (26). A recent study shows the T cell numbers are negatively correlated to serum IL-6, IL-10 and TNF-α concentration, but T cells will restore when IL-6, IL-10 and TNF-α concentrations are decreased in the disease resolution period (27). During acute stage of COVID-19, SARS-CoV-2 specific CD8^+^ T cells exhibit higher levels of IFN-γ, Granzyme B, Perforin, which are associated with enhanced cytotoxic effector functions (28). CD4^+^ T cells own the capacity to differentiate into differently functional subsets including Th1, Th2, Th17, Treg and T follicular helper cells (Tfh) (29). Virus-specific CD4^+^ T cells commonly contains Th1 and Tfh. Th1 plays antiviral role through secreting IFN-γ and other cytokines (30). Tfh participates in germinal center formation and sensitizes B cells to produce neutralizing antibody during SARS-CoV-2 infection (31). Age, gender, racial and/or pre-existing immunity may all account for the heterogeneity of COVID-19 patients and influence the disease outcomes (32). Differences in Treg status may be related to the heterogeneity of COVID-19. So, it is meaningful to focus on the change of FOXP3^+^ Tregs in the COVID-19 patients with different severity.

## Function and Mechanism of Treg: More Than Immunosuppression

Tregs are a subset of CD4^+^ T cells with immunosuppressive function and FOXP3 is the specific transcriptional factor of this subset. CD4^+^ CD25^+^ FOXP3^+^ Tregs account for approximately 10% of CD4^+^ T cells in human peripheral blood (33). FOXP3^+^ Tregs are critical for keeping immune tolerance (34). Sakaguchi lab showed that, transfer of suspensions of CD25^+^ population-depleted T cells into athymic nude mice produced autoimmune disease, while co-transfer with CD4^+^ CD25^+^ T cells prevented the autoimmune disease (2). Currently, FOXP3^+^ Tregs are also proved to play essential role in maintaining fetal-maternal tolerance (35), oral tolerance (36), transplantation tolerance (37) and even mucosal tissue tolerance (38).

**Figure 1:**
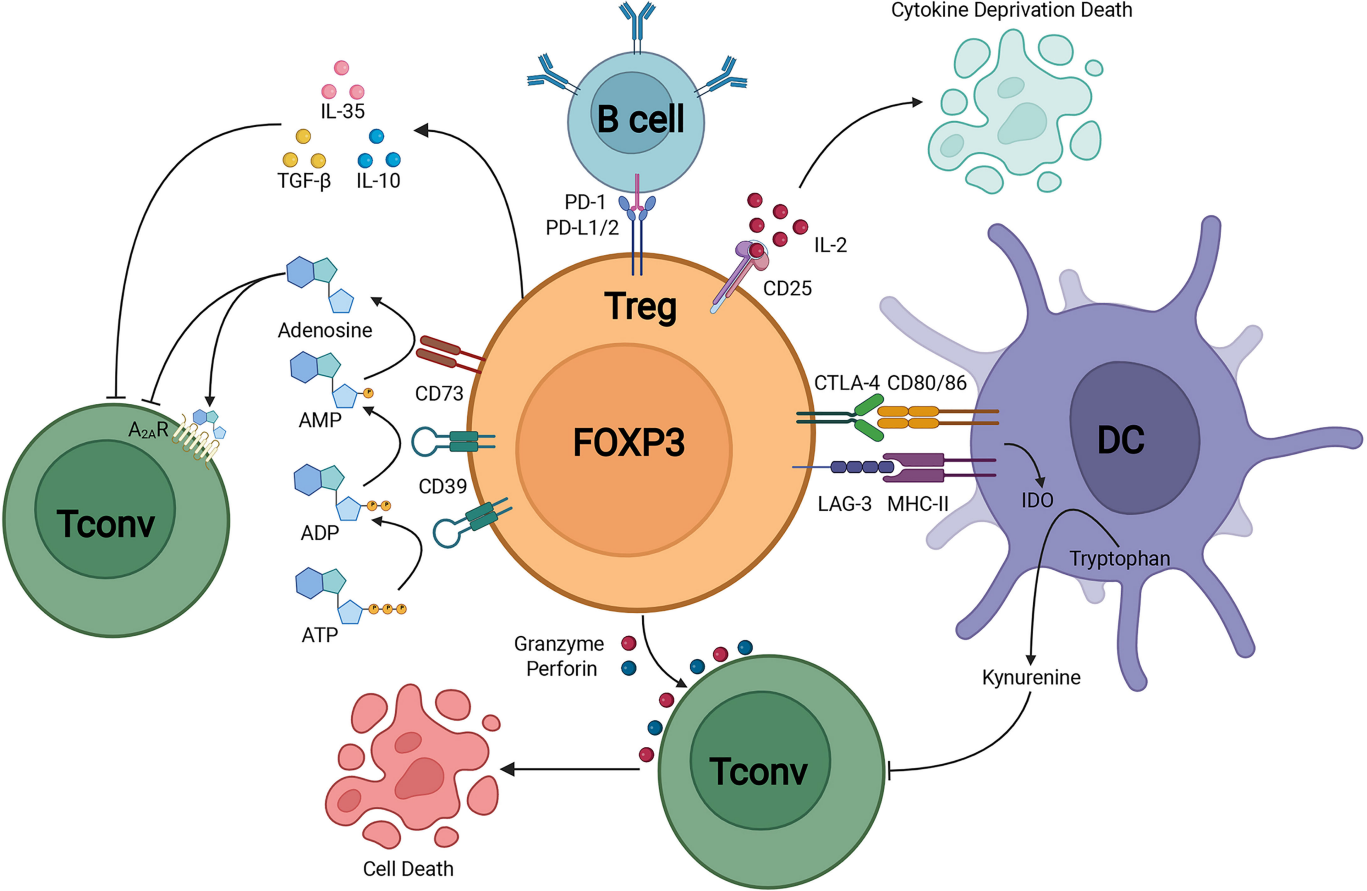
Treg suppressive mechanisms.Treg could secrete immunosuppressive cytokines including IL-10, TGF-β, and IL-35. In addition, Treg could induce cytolysis by Granzyme-dependent and Perforin-dependent mechanisms. The CD25 on Treg competitively binds to IL-2 and mediates apoptosis of effector immune cells through cytokine-deprivation. The interaction of LAG-3 and MHC-II mediates suppression of DC maturation, the interaction of CTLA-4 and CD80/86 mediates the production of immunosuppressive molecule IDO in DC. CD39 and CD73 facilitate the generation of pericellular adenosine, whose suppressive function is mediated through A_2A_R. Treg inhibits B cell activity *via* the interaction of PD-L1/2 and PD-1.

FOXP3^+^ Tregs keep the immune homeostasis *via* multiple mechanisms (**Figure 1**). Tregs could secrete inhibitory cytokines including IL-10, IL-35 and TGF-β (39). Furthermore, FOXP3^+^ Tregs highly expressed CD25 to compete for endogenous IL-2 and induced cytokine deprivation-mediated apoptosis of effector cells (40). Tregs also expressed CD39 which degraded ATP to AMP and inhibited the maturation of dendritic cells mediated by ATP (41). Co-expression of CD39 and CD73 on Tregs could convert ADP into adenosine, and adenosine bound to the adenosine A_2A_ receptor of effector T cells and thus inhibited the activation of effector T cells (42). The activation of adenosine A_2A_ receptor promoted the production of TGF-β while decreased the expression of IL-6, promoting the generation of Tregs (43). Tregs could downregulate the expression of costimulatory molecules CD80 and CD86 on dendritic cells (44). The cytotoxic T-lymphocyte-associated protein 4 (CTLA-4) expression on Tregs led to the reduction of CD86 through transendocytosis and impaired the activation of antigen-presenting cells (APCs) (45). Furthermore, *via* the CTLA-4 induced signaling, Tregs could upregulate the expression level of indoleamine 2,3-dioxygenase in dendritic cells, leading to the starvation of effector T cells (46, 47). Moreover, Tregs expressed lymphocyte activation gene 3 (LAG-3) which competitively bound to major histocompatibility complex class II (MHC-II) and restrained the maturation of dendritic cells (DCs) (48). Another important mechanism of Treg-mediated suppression was the Granzymes-dependent and Perforin-dependent cytolysis of CD8^+^ T cells and nature killer cells (49). As for B cells, besides the apoptosis through Perforin and Granzymes, the binding between programmed death-1 ligands (PD-L1) of Tregs and programmed death-1(PD-1) of autoreactive B cells impaired the proliferation and function of autoreactive B cells (50).

**Figure 2:**
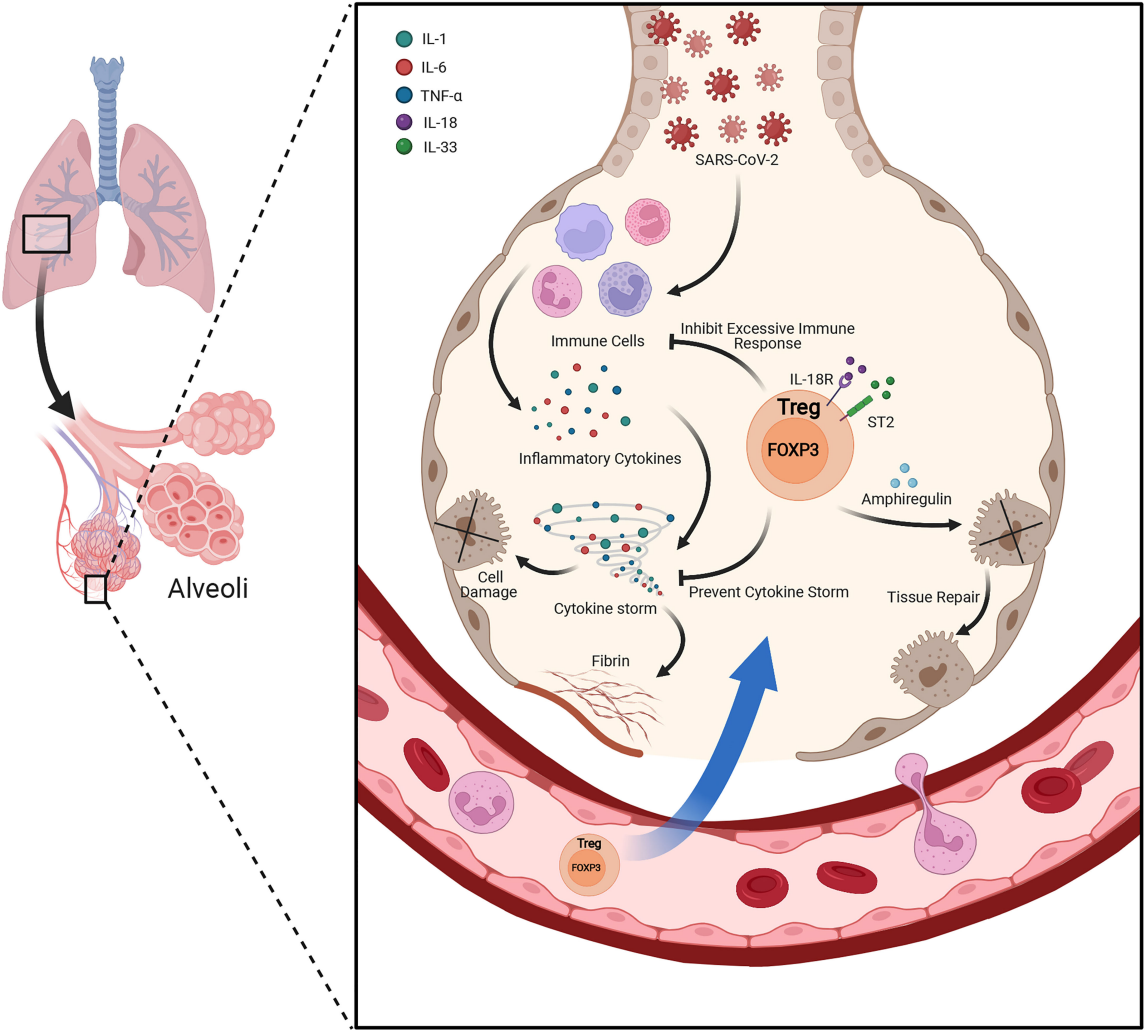
Lung resident Treg plays protective role under acute lung injury. Besides suppressing the excessive inflammatory response and keeping immune tolerance under the condition of acute lung injury, lung resident Tregs also produce Amphiregulin under the stimulation of IL-18 or alarmin IL-33 during lung injury by viral infection. Amphiregulin is crucial for Treg cell-mediated tissue repair.

FOXP3^+^ Tregs not only play essential role in the maintenance of immune homeostasis, but also play an indispensable role in regulating tissue homeostasis, inflammation and repair when resident in non-lymphoid tissues (51). The tissue resident Tregs own diverse functions, appeared in multiple sites including adipose tissue, cardiac muscle, skeletal muscle, lung, liver, central nervous system, skin and other tissues (51, 52). Recently we have revealed that insulin signaling induced the transition of visceral adipose tissue Tregs from CD73^hi^ST2^lo^ subset into a CD73^lo^ST2^hi^ subset through the HIF-1α/Med23-PPARγ axis and thus influenced beige fat production (53). The lung-resident Tregs are responsible for keeping immune tolerance and tissue repair (**Figure 2**). By using an influenza virus infection model, the researchers unveiled memory Tregs offered protective function during secondary encounters with pathogens (54). Another study of influenza virus infection showed at the early stage of lung injury, under the stimulation of IL-18 or alarmin IL-33, a group of IL18R^+^ Tregs would expand and produce tissue-repair protein Amphiregulin under a TCR independent manner (55). Moreover, Helios^+^ Treg cells were selectively recruited to lung tissues after influenza virus infection and enhanced the suppressive function on virus specific CD8^+^ T cells (56). Interestingly, when Tregs co-cultured with primary type II alveolar cells (AT2), AT2 cell proliferation directly increased *via* a CD103-dependent manner (57). However, during acute lung injury, the increased high-mobility group box 1 (HMGB1) impaired the expression of FOXP3 and CTLA-4 in lung resident Tregs through TLR4 and reduced Tregs immunosuppressive function, accelerating the lung injury (58).

Post-translational modifications (PTMs) of FOXP3 influence the stability and function of FOXP3, thereby altering the function and plasticity of Tregs (59). We have also unveiled some PTMs of FOXP3 in our previous work. Under the stimulation of lipopolysaccharide (LPS) and pro-inflammatory cytokines, Treg function was disrupted due to the K48-linked polyubiquitination degradation of FOXP3 by E3 ubiquitin ligase Stub1 (60). In contrast, E3 ubiquitin ligase MDM2 enhanced the Treg suppressive function through stabilizing FOXP3 in mouse (61). Ubiquitination of FOXP3 is reversible so deubiquitination is also critical for the function of FOXP3. USP21 prevented FOXP3 from proteasomal degradation through deubiquitination and enhanced FOXP3 stability, thus maintaining the expression of Treg signature genes (62). Additionally, we also proved USP44 stabilized FOXP3 by removing K48-linked ubiquitin modifications and maintained function of Tregs (63). Phosphorylation is another PTM of FOXP3, we demonstrated PIM1 kinase phosphorylated FOXP3 at S422 in human Tregs, and PIM1-specific inhibitor enhanced Treg suppressive activity by promoting FOXP3 DNA binding activity (64). Moreover, we found Kaempferol could enhance the suppressive function of Tregs *via* inhibiting the PIM1-mediated FOXP3 phosphorylation and increased protein level of FOXP3 (65). Similarly, small molecule inhibitor of PIM2 kinase also enhanced the function of Tregs by decreasing the phosphorylation of FOXP3 (66). PARP-1 negatively regulated the suppressive function of Tregs *via* FOXP3 poly(ADP-ribosyl)ation, PARP-1 inhibitors could promote FOXP3 stabilization and enhance Treg suppressive function (67). Besides the PTMs of FOXP3, the interaction of DBC1 and FOXP3 promoted the degradation of FOXP3 and weakened the suppressive function of Tregs (68). In conclusion, partner proteins or PTMs of FOXP3 are critical for Treg stability, plasticity and functions. Therefore, small molecules targeting the PTMs or partner-interactions of FOXP3 have the potentiality to be developed into drugs for COVID-19 therapy.

## The Change of Treg in SARS-CoV-2 Patients: A Controversial Topic

Previous study has shown that upon acute lung injury (ALI), lung-infiltrated Tregs are strongly induced to resolve lung injury and offer tissue repair (69–71). So, under the violent cytokine storm and lung epithelium damage caused by COVID-19, Tregs might be required to prevent tissue injury.

Several studies have reported the potential relationship between Treg and COVID-19 severity. Some research found the phenomenon of increasing proportion of Tregs or higher expression of Treg functional markers. For instance, a study revealed that higher proportion of CD25^+^ FOXP3^+^ Tregs among CD4^+^ T cells, increased mean fluorescence intensity (MFI) of FOXP3 and higher expression of activated Treg markers like KLRG1 and PD-1 in severe COVID-19 patients which all reverted to the baseline in the recovered patients (72). Similarly, another study demonstrated that, in critical COVID-19 patients, the frequency, the proliferation as well as the protein abundance of FOXP3, CTLA-4, GITR and ICOS of CD25^+^ CD127− FOXP3^+^ Tregs enhanced, along with their increasing suppressive function (73). In the bronchoalveolar lavage fluid (BALF) of COVID-19 ARDS patients, researchers found elevated Tregs and Th17 cells while declining T-cell populations (74). Interestingly, proportion of CD25^+^ CD127^−^ Tregs among the total CD4^+^ T cells and expression of CTLA-4 on Tregs increased in the prolonged SARS-CoV-2 positivity patients, compared with clinical recovery cohort and healthy donor cohort (75). Notably, the change of Tregs mainly reflected in the ascending percentage but not the absolute numbers, and COVID-19 patients were characterized by increasing percentage of naïve Tregs (CD45RA^+^ CCR7^+^) and central memory Tregs (CD45RA^−^ CCR7^+^) with robust expression of PD-1 (76). In addition, on 5 days post-infected (dpi), the CD4^+^ FOXP3^+^ Tregs of lung and PBMC exhibited an increasing trend in the nonhuman primate model of COVID-19 progression (77). Increase of the cell proportion and functional markers’ abundance possibly bring stronger suppression function of Tregs, but more research is required for this hypothesis.

The proportion change of Tregs in COVID-19 is still controversial, possibly due to the biphasic roles of Tregs during the process of viral infection. Several studies have reported the decrease of Tregs in the COVID-19 patients. For instance, a study reported that, compared with non-ICU hospitalized groups, the frequency of Tregs of ICU hospitalized patients was markedly decreased along with the Th17/Treg ratio significantly increased, the researchers also found the inhibitory function of the Tregs from ICU patients was impaired through the suppression assay (78). Another study also reported similar increase of Th17/Treg ratio in COVID-19 patients’ PBMC, which was related to poorer prognosis and lower abundance of Treg-relevant cytokines like IL-10 and TGF-β (79). Moreover, a single-cell transcriptomic analysis of viral antigen-reactive CD4^+^ T cells from 40 COVID-19 patients got the conclusion that SARS-CoV-2-reactive Tregs were dramatically reduced in hospitalized COVID-19 patients, while the proportions of cytotoxic follicular helper cells and cytotoxic T helper cells responding to SARS-CoV-2 were increased (80). Other study highlighted that, compared to mild or moderate subjects, the absolute number of total lymphocytes of severe COVID-19 patients was reduced and the amount of Tregs was negatively correlated to viral load, suggesting reduced Tregs stood for increased risk of worsening during the hospitalization (81). A study in Wuhan showed severe COVID-19 patients presented decreased regulatory T cells (CD3^+^ CD4^+^ CD25^+^ CD127^low^) proportion (82). Another study, taking advantage of CyTOF to analyze PBMCs of COVID-19 patients, reported that Tregs ratio increased during the progression from mild to severe condition but declined during the progression to critical condition, suggesting there was a dynamic change of Tregs during the progression of COVID-19 (83). Interestingly, a study analyzed the transcriptomes of CD4^+^ T cells of COVID-19 patients and found out CD25 was significantly upregulated in CD4^+^ T cells, but the proportion of FOXP3^+^ cells in CD4^+^ CD25^+^ T cells of severe patients is significantly reduced compared with moderate patients, the increasing expression of CD25 was correlated to the upregulation of FURIN which could promote the invasion of SARS-CoV-2 into lung epithelial cells (84). In the children infected with SARS-CoV-2, Tregs apparently declined during the acute phase and reverted to the baseline when recovered (85). Interestingly, a high-dimensional flow cytometry analysis of the severe COVID-19 airway indicated reduced Treg frequency compared with healthy control (86), suggesting that the function of lung resident Tregs was possibly impaired in severe COVID-19 cases. It has been known that Tregs could lose its stability *in vitro* under the stimulation of pro-inflammatory cytokines like IL-6 (87). So, under the inflammatory environment caused by COVID-19, abundant IL-6, IL-1 and IL-23 might induce the expression of RORγt and downregulate FOXP3 (88), leading to the reduction of Tregs in COVID-19 patients.

The variability of the Treg subsets composition was also reported in COVID-19 patients. For example, a study reported that the ratio of CD39^+^ Tregs in PBMCs was increased with disease severity in adult patients while the CD39^+^ Tregs were decreased in juvenile patients in an age-dependent manner (89). Another study showed the total Tregs had no change but only CCR4^Hi^ Tregs in the hospitalized COVID-19 cases were increased (90). Compared with moderate COVID-19 cases, the severe cases showed significantly lower proportion of CD45RA^+^ naive Tregs while a slightly higher proportion of CD45RO^+^ memory Tregs (91), suggesting the proportion of Treg subsets might predict the outcome of patients. A similar phenomenon was also observed in another study, especially in those with extremely severe COVID-19 compared with mild patients (92).

It was reported lymphopenia was an effective predictor for the patients suffered with COVID-19, lower blood lymphocyte percentage indicated poorer outcome (26). There might exist several potential mechanisms contributing to lymphopenia. The SARS-CoV-2 RNA was also detected in immune cells through single cell RNA-seq (93), SARS-CoV-2 might own the capacity to infect Treg through ACE2-independent receptors (94).

## Potential Treg-Based Therapy in COVID-19

Since Tregs have roles in keeping immune homeostasis and offering tissue repair, targeting and regulating the function of Tregs might be a good way for COVID-19 treatment. Cytokine storm and extensive lung damage caused by increased amounts of proinflammatory cytokines were particularly associated with disease severity. Although currently there is no specific therapeutic agent for the human coronavirus disease, some drugs with broad-spectrum antiviral activity or immunotherapies targeting dysregulated immune responses have been successfully used. Antiviral drugs such as remdesivir, favipiravir, ribavirin, as well as chloroquine have been reported to block SARS-CoV-2 infection and are undergoing clinical studies (95, 96). Treg-based therapies have been successfully used in treating autoimmune diseases and solid organ transplantation and have received initial success, aiming to ameliorate autoimmunity and restore immune tolerance. There is evidence that therapy with adoptive transfer of Tregs is helpful for autoimmune patients (97). This transfer of Tregs has been demonstrated to resolve fibroproliferation in an animal model of lung injury (71). IL-10 produced by Tregs plays an antifibrotic role and significantly contributes to the inhibition on fibroproliferation (98), suggesting that targeting Tregs could be a potential strategy to treat pulmonary fibrosis in severe COVID-19 patients. Currently, scientists are exploring to treat ARDS patients by infusion with umbilical cord-derived, allogeneic, ex-vivo expanded polyclonal CD4^+^ CD25^+^ Tregs (ClinicalTrials.gov Identifier: NCT05027815). Plenty of clinical trials working over the efficacy of Treg in COVID-19 are ongoing, some of which have achieved promising clinical effects, such as reduced lung inflammation (99) (ClinicalTrials.gov Identifier: NCT04468971). Circulating Tregs frequencies in severe COVID-19 patients were reduced (79, 80, 82), and the loss of Treg function could lead to a lung hyperinflammatory response. Therefore, increasing Tregs in blood by the infusion of ex-vivo expanded Tregs could suppress excessive inflammatory response in the lung and alleviate lung injury.

Besides the treatment by infusion of exogenous Tregs, raising Tregs *in vivo*, such as treatment with cytokines or small molecule drugs, could be another solution for the treatment of COVID-19 patients. IL-2 is a key survival factor required for the proliferation and inhibitory functions of Tregs by regulating the expression of FOXP3 and Treg secreted cytokines (100, 101). IL-2 binds to CD25 which is highly expressed on the cell surface of Tregs, activates STAT5 signaling and then influences the activity of Tregs (101). Low-dose IL-2 therapy has been used in the clinical treatment of autoimmune diseases such as systemic lupus erythematosus (SLE), rheumatoid arthritis (RA) and T1D and achieved excellent effects (100, 102–104). As IL-2 contributes to the development of both Tregs and Th17 cells, treatment with low concentrations of IL-2 might resolve the Treg/Th17 imbalance in COVID-19 patients (79, 101, 105). Actually, a clinical trial with low-dose IL-2 for SARS-CoV-2-related ARDS was completed last year (ClinicalTrials.gov Identifier: NCT04357444). Additionally, a phase 3 clinical trial has been registered recently for cytokines therapy on COVID-19 patients, evaluating the efficacy and safety of treatment with IL-2 or inhibitor of IL-17 (ClinicalTrials.gov Identifier: NCT04724629). However, the clinical observation showed that severe and critical patients had higher level of soluble IL-2R, which became a biomarker for early identification of severe COVID-19 and for predicting the clinical progression (106–108). The increased levels of soluble IL-2R could potentially scavenge IL-2, suggesting low-dose IL-2 therapy was not the optimal regimens for COVID-19 treatment (109). It is reported that an anti-human IL-2 (hIL-2) antibody can increase the Treg/Teff (effector T cells) ratio when bound to hIL-2 (110). A particular IL-2 monoclonal antibody JES6-1 brought selective expansion of Tregs and inhibited inflammation in mice experimental autoimmune encephalomyelitis (EAE) model (111). The imbalance of Tregs versus other immune cells *in vivo* results in autoimmune diseases and inflammatory. Ruxolitinib, a JAK 1/2 inhibitor, was reported to decrease the frequency of Th17 while increase FOXP3 abundance and Treg frequency (112). A Phase I/II trial for COVID-19 treatment with Ruxolitinib was completed in 2021(ClinicalTrials.gov Identifier: NCT04334044). It’s reported that transient breakdown of Treg tolerance can activate DCs and induce DCs’ protective adaptive immunity against SARS-CoV-2 (113), so regulation on Tregs and DCs might be a promising way for COVID-19 therapy.

Moreover, some small-molecule drugs which could promote the function of Tregs might be used to resist the cytokine storm caused by COVID-19. From our laboratory previous study, we found GSK3 inhibitor SB216763 could enhance the suppressive function of human iTregs by promoting IL-10 production and decreasing proinflammatory iTregs (114). Another study suggested GSK3 inhibition as a potential therapeutic approach against SARS-CoV-2 (115). PI3K-Akt-mTOR signaling axis was critical for the development of Tregs (116). Rapamycin, an inhibitor of mTOR used in the therapy of T1D patients, promote the expansion of Tregs while inhibit the proliferation of effector T cells (117, 118). Rapamycin treatment obtained the potential to prevent the cytokine storm in patients with severe COVID-19 (119). All-trans retinoic acid (atRA), a metabolite of vitamin A, was able to facilitate the differentiation of Tregs from naïve CD4^+^ T cells and suppressed the *de novo* generation of Th17 from naïve CD4^+^ T cells (120, 121). Besides regulating the balance of Treg/Th17, atRA could maintain the stability and function of nTregs under inflammatory environment (122). AtRA was also reported to exhibit antiviral effect against SARS-CoV-2 by inhibiting 3CLpro activity (123).

Antigen-specific TCR, which could be redirected towards a desired antigen, is an option for Treg-based therapy (124). TCR-Tregs were able to be expanded ex-vivo and functioned more efficiently than polyclonal Tregs in animal models of T1D, RA and transplantation (125–127). Targeting specific antigen of SARS-CoV-2, TCR-Treg therapy could have advantages of lower dosage but higher efficiency and own the therapeutic potential in COVID-19 patients. Another strategy is CAR-Tregs, which have the ability to bind to tissue-specific autoantigens and specifically focus the suppressive functions on the diseased site (128). CAR-Treg therapy has been demonstrated to work well in various preclinical models including EAE, colitis and experimental allergic asthma (129–131); and one Phase I/II trial on CAR-Treg therapy in renal transplantation is ongoing (ClinicalTrials.gov Identifier: NCT04817774). Although CAR-Treg therapy attracts much attention in a variety of autoimmune diseases and tumor, it hasn’t been used in COVID-19 treatment yet. The ability of CAR-Treg to induce immunological tolerance provides the potential applications of CAR-Treg in SARS-CoV-2 treatment.

CTLA-4 is a functional marker of Tregs. It interacts with CD80 and CD86, two ligands of stimulatory receptor CD28. By increasing trans-endocytosis and degradation of two ligands, CTLA-4 reduces co-stimulatory signals for T-cells (45). The recombinant Fc-fused CTLA-4 protein, Abatacept has been reported to interfere with T-cell signaling and activation, and therefore it has been used for several years for the immunotherapy of many autoimmune diseases (109). Recently, a clinical trial which uses Abatacept in the therapy of COVID-19 patients has been completed (ClinicalTrials.gov Identifier: NCT04593940). Researchers from University of Alabama also have registered a clinical trial for using Abatacept with COVID-19 vaccination in the therapy (ClinicalTrials.gov Identifier: NCT05080218). An epidemiological survey indicated that Abatacept, one of targeted biologic and synthetic disease modifying anti-rheumatic drugs (tDMARDs), could decrease incidence of COVID-19 and bring milder symptoms (132). This provided the basis for using CTLA-4-based therapy in COVID-19 patients. In addition to CTLA-4, TGF-β, a Treg-derived immunoregulatory molecule, is also believed as a target for SARS-CoV-2 treatment. TGF-β could cause the lung fibrosis and be involved in the fluid homeostasis in the lung (133). Therefore, blocking TGF-β by neutralization and elimination TGF-β with antibodies and/or TGF-β inhibitors becomes a promising approach to protect lungs from the development of fibrosis (134).

A cohort of research on COVID-19 patients showed that expression of Notch4 was increased on Tregs and associated with disease severity, mortality, and recovery. Notch4-amphiregulin nexus was identified as an assumed target of therapy in viral respiratory infections, including SARS-CoV-2 and influenza (135). Pathway of Notch4 and Notch ligand delta-like ligand 4 (DLL4) is shown to increase H3K4me3 around the *foxp3* locus to stabilize FOXP3 expression, which further regulates the differentiation and function of Tregs (136). These studies suggest that the interference along the Notch4-DLL4 axis might be a feasible treatment strategy for relieving of COVID-19. Furthermore, the dual roles of FOXP3^+^ Tregs during viral infection should be reminded. The dynamic changes of Tregs including proportion, suppression function and FOXP3 stability under different COVID-19 stage should be well uncovered before Treg-based therapy.

## Conclusion and Prospective

Currently, the changes on the proportion and absolute number of Tregs under SARS-CoV-2 infection remain debatable. Several possibilities exist for causing these controversial results. Firstly, the marker to identify Treg is not unified in different studies, researchers use different markers to define Tregs. Secondly, the patients in the studies are possibly infected by different variants of SARS-CoV-2, different variants vary in pathogenicity and induce different degree of immune response, but most researchers did not distinguish which variants infected patients. Thirdly, in different studies, there was no criteria to define the severity order of patients or to identify the subset of Tregs. Fourthly, the change of Tregs during COVID-19 is possibly dynamic, patients might stay at different stage of COVID-19. Besides the changes on proportion and absolute number of Tregs in COVID-19 patients, possibly the change on the suppressive function (stability, plasticity etc.), the composition of Treg subsets and tissue-specific subsets should be more noteworthy.

**Figure 3:**
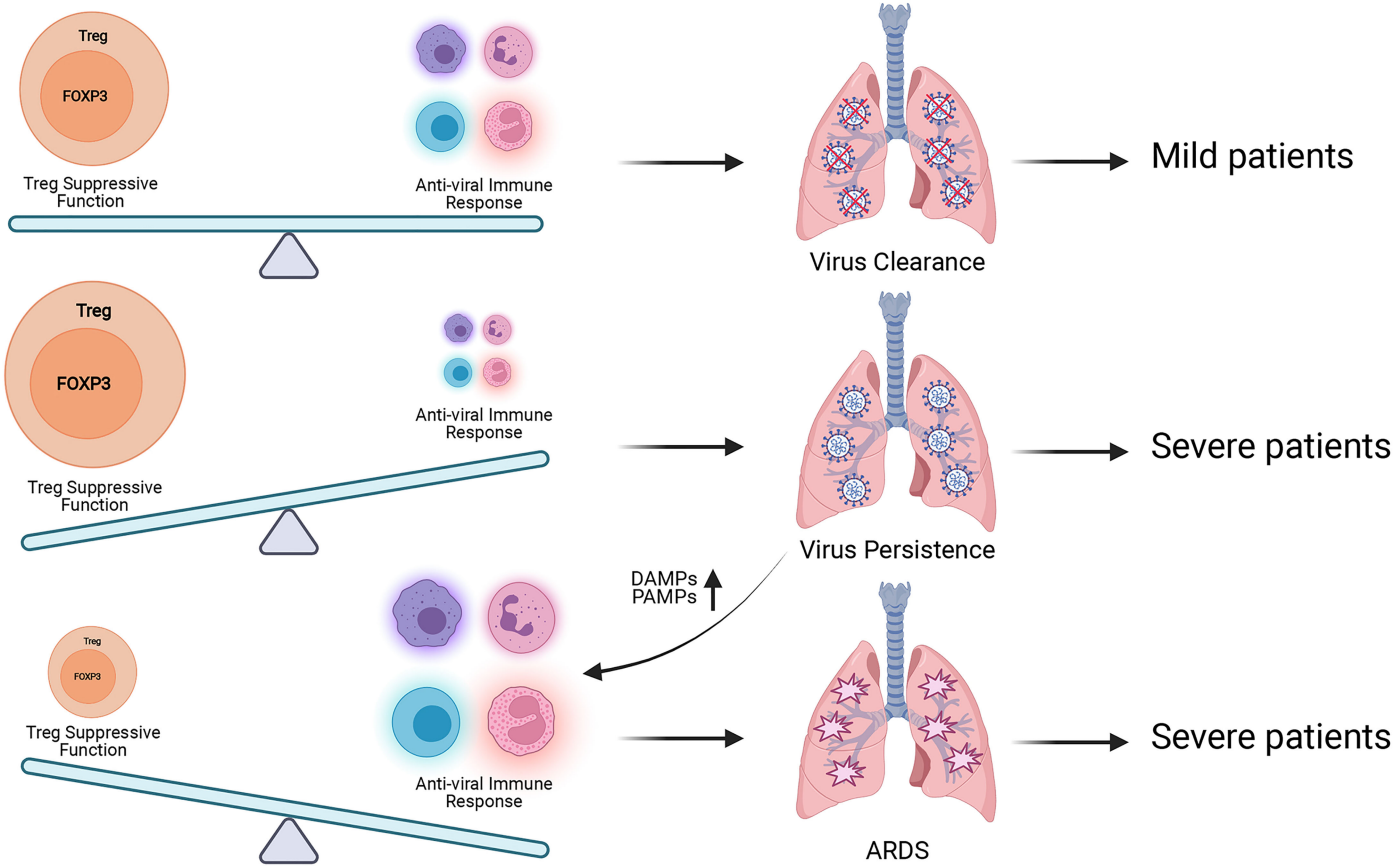
Treg has dual roles during SARS-CoV-2 infection. The balance between Treg suppressive function for maintaining immune homeostasis and antiviral immune responses for eliminating SARS-CoV-2 is proposed to be kept well in the mild patients. When this balance is broken, patients will suffer poorer prognosis. Excessive Treg activity will result in virus persistence, more production of pro-inflammatory DAMPs and PAMPs and exacerbated inflammation. Insufficient Treg activity will contribute to aggressive inflammatory response with cytokine storm which leads to tissue damage and ARDS. More researches are required to explore the best time for Treg-based therapy to avoid severe COVID-19 symptom.

The dual roles of FOXP3^+^ Treg during antiviral immune responses should also be noteworthy (**Figure 3**). It is supposed that, in the mild cases there exists an ideal balance between the suppression of FOXP3^+^ Treg and antiviral immune response, body can eliminate SARS-CoV-2 while maintaining immune homeostasis. If the suppressive function of FOXP3^+^ Tregs is too strong at the early stage of virus infection, the immune response of host will be suppressed by Tregs and cannot produce an adequate immune response for viral clearance. Then the virus will speed up its replication and produce more PAMPs (Pathogen-associated molecular patterns). The tissue injury caused by viral infection produces more DAMPs (Damage-associated molecular pattern), and activates acute immune response, leading to severe cases. When the immune responses caused by SARS-CoV-2 are over-activated, which exceed the suppressive control of FOXP3^+^ Tregs, the excessive immune responses will contribute to tissue damage and trigger cytokine storms, leading to severe cases. Based on this hypothesis, the introducing time and efficacy of Treg-based therapy should be critical for COVID-19 treatment, and we hope Treg-based therapy can help more COVID-19 patients avoid severe cases.

## Author Contributions

ZX and XJ contributed equally to writing the manuscript. BL and XD participated in reviewing and critically correcting the manuscript. All authors contributed to the article and approved the submitted version.

## Funding

This research is supported by National Key Research and Development Program of China 2019YFA09006100; National Natural Science Founding of China grants 32130041, 81830051 and 31961133011; Innovative research team of high-level local universities in Shanghai and Shanghai Collaborative Innovation Center of Cellular Homeostasis Regulation and Human Diseases; Key Laboratory of Cell Differentiation and Apoptosis of Chinese Ministry of Education, Shanghai Frontiers Science Center of Cellular Homeostasis and Human Diseases; Shanghai Jiao Tong University (SJTU) - The Chinese University of Hong Kong (CUHK) Joint Research Collaboration Fund and the Fundamental Research Funds for Central Universities.. The principal recipient of all the fundings above is BL, Ph. D.

## Conflict of Interest

The authors declare that the research was conducted in the absence of any commercial or financial relationships that could be construed as a potential conflict of interest.

## Publisher’s Note

All claims expressed in this article are solely those of the authors and do not necessarily represent those of their affiliated organizations, or those of the publisher, the editors and the reviewers. Any product that may be evaluated in this article, or claim that may be made by its manufacturer, is not guaranteed or endorsed by the publisher.
